# Corneal Biomechanical Properties of Keratoconic Eyes Following Penetrating Keratoplasty

**DOI:** 10.4274/tjo.79664

**Published:** 2018-09-04

**Authors:** Hamidu Gobeka, Özlem Barut Selver, Melis Palamar Onay, Sait Eğrilmez, Ayşe Yağcı

**Affiliations:** 1 Ege University Faculty of Medicine, Department of Ophthalmology, İzmir, Turkey

**Keywords:** Corneal biomechanical properties, Ocular Response Analyzer, keratoconus, penetrating keratoplasty

## Abstract

**Objectives:**

To investigate the corneal biomechanical properties of keratoconic eyes following penetrating keratoplasty (PKP).

**Materials and Methods:**

Thirty-five patients (70 eyes) were enrolled to this prospective study. Operated and contralateral keratoconic eyes were defined as Group 1 and 2, respectively. All patients underwent ophthalmological examination and measurements of corneal biomechanical properties by Ocular Response Analyzer (ORA), intraocular pressure (IOP) by Goldmann applanation tonometry, and central corneal thickness (CCT) by Pentacam. Shapiro-Wilk W test was performed to test normality of the data. The statistical significance was evaluated with the paired t-test and Wilcoxon signed ranks test. Pearson correlation and Spearman rho tests were used for correlation analysis.

**Results:**

The average age and male/female ratio were 31.34±11.65 (15-60) years and 21/14, respectively. The mean values of the data obtained from Group 1 and 2 respectively were: corneal hysteresis (CH): 9.35±1.66, 8.18±1.84 mmHg (p=0.013), corneal resistance factor (CRF): 9.48±1.96, 7.14±2.05 mmHg (p<0.001), IOPcc: 16.90±4.32, 14.26±3.69 mmHg (p=0.004), IOPg: 15.45±4.61, 10.91±3.97 mmHg (p<0.001), IOPapl: 14.26±3.11, 13.09±2.54 mmHg (p=0.046), and central corneal thickness (CCT): 545.64±60.82, 442.60±68.14 μM (p<0.001). The positive correlation between CH and CRF was moderate (r=0.444) in Group 1 and strong (r=0.770) in Group 2. There was a moderate negative correlation between CH and IOPcc in both groups (r=-0.426, r=-0.423), but CH was not correlated with IOPg or IOPapl in either group. There were weak to strong positive correlations between CRF and all IOP values in both groups. There was no correlation between CRF and CCT in Group 1 (r=0.075) and a very weak correlation in Group 2 (r=0.237). Only IOPcc and IOPg were strongly correlated in both groups.

**Conclusion:**

Better understanding of corneal biomechanical properties is essential for elucidating the pathophysiology and diagnosis of several corneal pathologies such as keratoconus. The biomechanical properties of keratoconic eyes seem to be closer to normal values after PKP.

## Introduction

The cornea has unique viscoelastic properties that enable it to deform under stress and then return to its original state. Collectively, these are called the biomechanical properties of the cornea. In recent years, the in vivo evaluation of corneal biomechanical properties has gained importance. Although new devices are under development for such analyses, the Ocular Response Analyzer (ORA) device is currently in widespread use.^1^

Since the introduction of this device into clinical practice, several studies have been conducted on the biomechanical properties of the cornea.^2^ These studies examine a wide range of findings, from demographic data to the effects of corneal surgeries and various corneal pathologies on corneal biomechanical properties.^1,2,3,4,5,6,7,8,9,10,11,12^

Keratoconus is a progressive degenerative disorder of the cornea which inevitably affects its biomechanical parameters. Moreover, corneal transplant by keratoplasty, the surgical treatment option for advanced keratoconus, results in another change in biomechanical parameters.^2^

In this study, we aimed to evaluate and compare corneal biomechanical parameters in both eyes of patients with bilateral keratoconus who underwent unilateral penetrating keratoplasty (PKP). We also examined the relationship between the results and patients’ demographic characteristics.

## Materials and Methods

In this study, we prospectively examined the corneal biomechanical properties of the operated and unoperated eyes of patients diagnosed with keratoconus who underwent unilateral PKP between 2013 and 2015 in the Cornea, Contact Lens, and Oculoplasty Unit in the Ege University Faculty of Medicine, Department of Ophthalmology. Eyes that underwent PKP were designated as Group 1 (study group) and the unoperated keratoconus eyes were designated as Group 2 (control group). The study was approved by the Ege University Faculty of Medicine, Clinical Research Ethics Committee. Voluntary informed consent forms were obtained from all patients.

Thirty-five patients aged 15-61 years were included in the study. All patients were diagnosed with keratoconus and underwent unilateral PKP surgery. Inclusion criteria included the absence of postoperative complications, absence of accompanying systemic (e.g. diabetes) or ocular diseases (e.g. glaucoma), no contact lens use, and no prior ocular surgery other than PKP (e.g. cataract surgery, LASIK).

The patients were examined no earlier than 15 days after the corneal sutures were removed. All patients underwent detailed ophthalmologic examination, best corrected visual acuity measurement, slit-lamp anterior segment examination, and posterior segment examination with a 90 diopter (D) lens following pupil dilation with 1% tropicamide. Intraocular pressure (IOP) was measured with a Goldmann applanation tonometer (Haag-Streit AG, Koning, Switzerland) (IOPapl) and an ORA was used to measure the following corneal biomechanical properties: corneal hysteresis (CH), corneal resistance factor (CRF), corneal compensated IOP (IOPcc), and Goldmann-correlated IOP (IOPg). The average of 4 measurements was recorded. In addition, central corneal thickness (CCT) was measured with a Pentacam (Oculus Pentacam version 1.20/10 Germany) device.

### Statistical Analysis

Data obtained in the study were statistically analyzed using SPSS (SPSS Inc., Chicago, IL, USA) version 16 for Windows software package. The conformity of the data to normal distribution was assessed with a Shapiro-Wilk W test. Parametric data conforming to normal distribution were evaluated in terms of statistical significance with a dependent t-test; data not conforming to normal distribution and other non-parametric data were evaluated using a Wilcoxon signed rank test. For correlation analysis, normally distributed parametric data were analyzed with Pearson correlation test while data not conforming to normal distribution and non-parametric data were analyzed with a Spearman rho test. Absolute correlation values of 0 to 0.25 were interpreted as very weak or no correlation, 0.25-0.50 as weak correlation, 0.50-0.75 as moderate correlation, and >0.75 as strong correlation. The intragroup consistency (reliability) of IOP measurements was assessed using an F-test. P values of <0.05 were considered statistically significant.

## Results

The mean age of the patients was 31.34±11.65 (15-60) years. The female to male ratio was 14:21 (2:3). Simple interrupted sutures were used in all procedures. Graft diameter was 7.75 mm in 23 patients (65.7%), 7.50 mm in 9 patients (25.7%), 8 mm in 2 patients (5.7%), and 9 mm in 1 patient (2.9%).

As expected, there was a significant increase in vision in all Group 1 eyes. Median pre- and postoperative visual acuity values were 1.3 (0.7-3.1) logMAR and 0.3 (0-1.5) logMAR, respectively (p<0.001).

The median interval between suture removal and ORA measurement was 10 months (0.5-492 months). All measured data (except for waveform score [WS]) conformed to normal distribution according to the Shapiro-Wilk W test.

The mean CH, CRF, IOPcc, IOPg, IOPapl, and CCT values of the groups are shown in Table 1. There were statistically significant differences between Groups 1 and 2 in all variables.

The median WS was 4.10 (2.20-7.50) in Group 1 and 5.10 (1.20-8.50) in Group 2 (p=0.376).

Analysis of correlations between age and corneal biomechanical parameters (CH, CRF, IOPcc, IOPg, and IOPapl) (Table 2) revealed no significant results other than very weak negative correlations between age and CH and CRF in Group 2 (r=-0.216, -0.242).

In the evaluation of gender differences in the corneal biomechanical properties of the eyes in Group 2, it was observed that females had higher CH (8.69 vs. 7.84 mmHg) and CRF (7.61 vs. 6.82 mmHg) values, but the differences were not statistically significant (p=0.186 and p=0.275, respectively).

As shown in Table 3, intragroup comparisons revealed a moderate positive correlation between CH and CRF values in Group 1 (r=0.444) and strong positive correlation in Group 2 (r=0.770). Moderate negative correlations were observed between CH and IOPcc in both Group 1 and Group 2 (r=-0.426, r=-0.423). There was no correlation between CH and IOPg, IOPapl, or CCT in either group. CRF and IOPcc were weakly or very weakly correlated in Group 1 (r=0.334) and Group 2 (r=0.178). CRF and IOPg showed a strong positive correlation in Group 1 (r=0.663) and a moderate positive correlation in Group 2 (r=0.575). There was a weak correlation between CRF and IOPapl in both Group 1 and Group 2 (r=0.277, r=0.298). CRF and CCT were not correlated in Group 1 (r=0.075), but showed a very weak correlation in Group 2 (r=0.237). There was a very strong positive correlation between IOPcc and IOPg in Group 1 (r=0.911) and a moderate positive correlation between them in Group 2 (r=0.771). Weak correlations were observed between IOPcc and IOPapl in both groups (r=0.357, r=0.371). There were also weak correlations between the IOPg and IOPapl values in both groups (r=0.362, r=0.384). CCT was not correlated with any corneal biomechanical parameter in Group 1, but was very weakly correlated with CRF (r=0.237) and IOPcc (r=0.487) and moderately correlated with IOPg (r=0.529) in Group 2.

In our evaluation of the consistency between IOP values, intragroup correlation coefficients (ICC) for IOPapl and IOPcc in Groups 1 and 2 were 0.276 (95% confidence interval [CI]=-0.027-0.543; p=0.022, F-test) and 0.330 (CI=0.019-0.588; p=0.019, F-test), respectively (Table 4). ICC values for IOPapl value and IOPg were 0.327 (95% CI=0.010-0.588, p=0.023, F-test) in Group 1 and 0.291 (CI=-0.013-0.556, p=0.019, F-test) in Group 2. For IOPcc and IOPg values, ICC values in Groups 1 and 2 were 0.866 (95% CI=0.563-0.947, p<0.0001, F-test) and 0.559 (CI=-0.073-0.828, p<0.0001, F-test), respectively.

## Discussion

Keratoconus is a degenerative process that causes changes in corneal biomechanical parameters. In eyes with keratoconus that undergo keratoplasty, another change in biomechanical parameters is expected.^2^

The reliability of the ORA device is the main factor in the ability to accurately evaluate corneal biomechanical parameters. Version 2.04 of the ORA includes WS as a scale of 0-10, with higher values corresponding to greater measurement reliability. In previous studies on the reliability of using ORA, Lam et al.^13^ recommended taking 3 measurements with WS ≥3.5, while Ehrlich et al.^14^ recommended a WS cut-off of 6.5. In another study, Mandalos et al.^15^ used a cut-off value of 6.0. Ayala and Chen^16^ recommended using measurements with WS of 7 or above whenever possible in order to increase reliability. In our study, the mean WS was 4.10 in eyes that underwent PKP and 5.10 in unoperated keratoconus eyes. These values are consistent with the reliability values reported by Lam et al.^13^ The low WS values in our study, particularly in the corneas that underwent keratoplasty, may be attributable to scar tissue at the recipient bed-graft junction in eyes that underwent keratoplasty and the presence of abnormal topographic changes in both groups. Detailed WS data were not provided in other studies with patient populations similar to ours, thus precluding comparison with other studies on this point.

Regarding the effect of sex on corneal biomechanical parameters, there are studies in the literature indicating no significant effect^17,18,19^ as well as studies reporting statistically significant differences.^20,21^ It is conceivable that sex differences in corneal biomechanical properties may vary between different ethnicities, explaining these conflicting results. Our evaluation of sex-based differences in the corneal biomechanical properties of unoperated keratoconus eyes in this study showed that CH and CRF values were higher in women, but that the difference was not statistically significant.

Investigation of the effect of age on corneal biomechanical parameters has revealed no clinically significant differences in many previous studies. In a study by Kamiya et al.^17^ involving 204 eyes of healthy individuals with a mean age of 46.7±19.4 years, a minimal but statistically significant negative correlation was observed between CH and CRF values, while Ortiz et al.^22^ reported significant differences in CH and CRF values only in individuals younger than 14 and older than 60 years of age. However, a linear correlation was not observed between age and these two biomechanical parameters. Kotecha et al.^23^ reported that CH decreased by 0.28 every decade, while Foster et al.^24^ observed that CH decreased by 0.34 and CRF value decreased by 0.31 with each decade of age. In our study, there was a negative correlation, albeit very weak, between age and CRF in unoperated eyes with keratoconus (r=-0.242). Due to the progressive nature of keratoconus, these findings may be related to age-related progression and the presence of more advanced disease in older patients. However, because normal corneas were not included in our evaluation of the effects of age and sex on CH and CRF values, we believe the sex differences observed in this study may not reflect those in the healthy population.

In keratoconus, corneal biomechanical properties are affected by various factors including collagen fibrils and the organization of the main corneal components and cells within the tissue.^25,26,27,28^ In previous studies concerning the biomechanical parameters of keratoconic eyes, it is reported that CH and CRF values are lower compared to normal corneas, and that this decrease is correlated with disease stage.^1,22,29,30,31,32,33^ Kirwan et al.^34^ examined corneal biomechanical properties in 3 groups (normal eyes, advanced keratoconus, and forme fruste [early] keratoconus [FFK]) and reported that CH and CRF values were significantly lower in eyes with keratoconus compared to FFK and normal eyes. Consistent with previous studies, we also found that the corneal biomechanical values of eyes with keratoconus (CH: 8.18±1.84 mmHg, CRF: 7.14±2.05 mmHg) were lower compared to literature data on normal healthy eyes (normal range, CH: 9.3±1.4-11.4±1.5 mmHg, CRF: 9.2±1.4-11.9±1.5 mmHg).^35^

The impact of keratoplasty on corneal biomechanical properties seems unavoidable.^3,5,6,7^ There are studies reporting that this effect differs in lamellar and PKP.^7,8,^^9^ The main factors that can contribute to changes in CH and CRF values after PKP are the biomechanical properties of the transplanted graft, the graft diameter, fibrotic wound healing at the graft-host junction, and the biomechanical properties of the recipient corneoscleral rim. While the first three factors have a positive effect on CH and CRF values, the presence of weak tissue from the keratoconic cornea in the corneoscleral rim of the recipient bed negatively affects CH and CRF values. In the relevant literature, Yenerel et al.^3^ reported that CH and CRF values were higher in eyes that underwent PKP compared to eyes with FFK or advanced keratoconus. It has also been shown that both CH and CRF approach values seen in normal eyes after PKP. Goldhagen et al.^36^ detected CH and CRF values close to those of normal corneas in keratoconus eyes that underwent PKP. Consistent with the literature, we observed in the present study that CH and CRF values were significantly higher after PKP when compared with unoperated keratoconus eyes (CH: p=0.013, CRF: p<0.001).

Strong correlation has been reported between CH value and CRF value, which are corneal viscoelastic parameters.^37^ Similarly, in the present study there was a moderate positive correlation between CH and CRF values in PKP eyes (r=0.444) and strong positive correlation in unoperated keratoconus eyes (r=0.770). The weaker correlation in eyes that underwent keratoplasty may be due to the effect of the fibrotic scar on cumulative values.

It is known that CH and CRF values are strongly correlated with CCT.^38,39,40,41^ Most studies in the literature have reported a strong positive correlation between CH and CRF values and CCT in normal eyes.^38,42,43,44,45,46,47^ Unlike these studies, Broman et al.^48^ observed different CH values in eyes with the same CCT value, which they attributed to the possible influence of other unidentified factors on corneal biomechanical properties.^34,49^ In our comparison of unoperated keratoconus corneas and keratoconus corneas that underwent PKP, we did not observe the correlation between CH and CRF values and CCT that exists in normal corneas, which supports the theory of multiple unidentified factors.

Studies investigating the relationship between CH and CRF values and IOP values have shown that CH is negatively correlated with IOPcc.^42,49,50,51,52^ This is likely due to the interaction between CH and CCT.^39^ As CCT increases, CH and the measured IOPg value also increase, while IOPg and IOPcc diverge.^38,42^ However, a study by Liu and Roberts^28^ demonstrated that the correlation between CCT and IOP values is not a simple linear relationship, but a complex and non-linear association. Furthermore, some believe that IOPcc value is a more accurate because it is obtained by eliminating the effect of CCT.^5^ Similar to the literature, correlation analysis between CH and CRF values and IOP values in our study revealed a moderate negative correlation between the CH value and the IOPcc value in both groups, but CH value was not correlated with IOPg or IOPapl values.

In the literature, CRF value is reported to be positively correlated with IOP values in healthy eyes.^53^ In parallel to existing data, we also observed positive correlations between CRF and all IOP values, ranging in strength from weak to strong, in both groups of eyes in our study. However, the correlations were weaker than those seen in healthy corneas.

The accepted strong positive correlations between CCT value and IOPapl and IOPg values are a result of the CCT increasing corneal resistance to applanation. Because IOPcc is obtained by correcting for the effect of corneal thickness on IOP, this value is least dependent on CCT.^54,55^ In our study, we detected no correlation between CCT and IOP values in either group. We believe that because our study involved eyes that underwent PKP and eyes with keratoconus, the relationship between biomechanical factors and IOP and CCT may have been affected by different variables than those reported in the literature.

Regarding consistency between IOP values, Ouyang et al.^56^ showed that in a normal population, repeated ORA IOP measurements are equivalent to IOPapl values and that ORA IOP values are valid and reliable. In our analysis of the intragroup consistency of measured IOP values, we did not observe significant agreement between IOPcc and IOPg values and IOPapl values in the eyes that underwent PKP or the keratoconus eyes. However, there was significant consistency between IOPcc and IOPg values in both groups. This is because IOPcc and IOPg values are both ORA IOP values and were obtained from the same device.

Aside from all these data, another factor that is likely to affect ORA parameters in eyes that undergo PKP is graft diameter. Corneal biomechanical results after keratoplasty using a large graft are reported to be closer to normal values. Large grafts have several advantages: there is less postoperative astigmatism due to a more peripheral graft-recipient interface, and the maximum amount of abnormal cornea is removed and replaced with normal donor tissue. In terms of graft biomechanical properties in keratoconus, a large graft may be expected to provide the best results and yield more stable postoperative refractive outcomes; however, grafts with large diameters (>8.5 mm) have certain limitations such as high graft rejection and failure rates.^9,57^ Therefore, we believe that when performing keratoplasty, these factors should be evaluated and graft diameter should be selected so as to bring the biomechanical properties of the cornea closer to normal while assessing the risks and benefits. The majority of the grafts used in the keratoplasty procedures in our study were 7.5 mm or 7.75 mm in diameter, which was not considered adequate data for a comparison of graft diameter. Therefore, we did not evaluate the effect of graft diameter on corneal biomechanical properties.

## Conclusion

In conclusion, while the biomechanical properties of eyes with keratoconus approach normal values after PKP, there are still important limitations to the comparison of these eyes using an ORA. Because measurements are taken 3-4 mm from the central cornea, keratoconic corneas with decentralized irregularity may be overlooked with this device and it may not be possible to evaluate response of the entire cornea in cases where the central 7-8 mm has been replaced, as in keratoplasty. In addition, central corneal surface irregularity and the presence of a corneal scar can interfere with the infrared specular reflection beam of the ORA, leading to a waveform change. Therefore, all of these potential limitations should be taken into consideration and the importance of reliability should not be overlooked in the ORA examination of all non-normal corneas.

## Figures and Tables

**Table 1:**
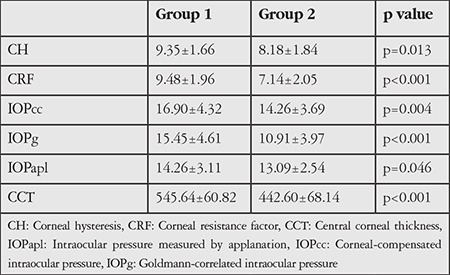
Comparison of mean ocular response analyzer corneal parameters in Groups 1 and 2

**Table 2:**
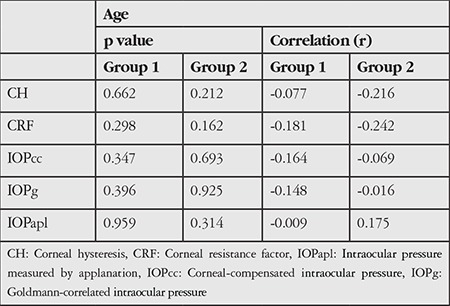
Analyses of statistical significance and correlation between age and Ocular Response Analyzer corneal parameters

**Table 3:**
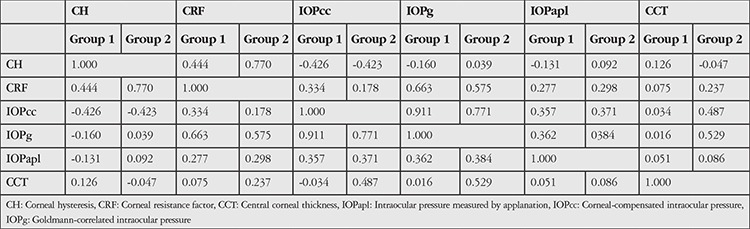
Intragroup correlations between corneal biomechanical parameters

**Table 4:**
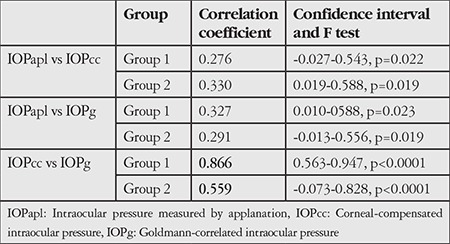
Consistency between intraocular pressure measurements within Groups 1 and 2
